# Involvement of STAT5 in Oncogenesis

**DOI:** 10.3390/biomedicines8090316

**Published:** 2020-08-28

**Authors:** Clarissa Esmeralda Halim, Shuo Deng, Mei Shan Ong, Celestial T. Yap

**Affiliations:** 1 Department of Physiology, Yong Loo Lin School of Medicine, National University of Singapore, Singapore 117593, Singapore; phsceh@nus.edu.sg (C.E.H.); phsdes@nus.edu.sg (S.D.); e0013225@u.nus.edu (M.S.O.); 2 Medical Science Cluster, Cancer Program, Yong Loo Lin School of Medicine, National University of Singapore, Singapore 117597, Singapore; 3 National University Cancer Institute, National University Health System, Singapore 119074, Singapore

**Keywords:** STAT5, cancer, metastasis, proliferation, angiogenesis

## Abstract

Signal transducer and activator of transcription (STAT) proteins, and in particular STAT3, have been established as heavily implicated in cancer. Recently, the involvement of STAT5 signalling in the pathology of cancer has been shown to be of increasing importance. STAT5 plays a crucial role in the development of the mammary gland and the homeostasis of the immune system. However, in various cancers, aberrant STAT5 signalling promotes the expression of target genes, such as cyclin D, Bcl-2 and MMP-2, that result in increased cell proliferation, survival and metastasis. To target constitutive STAT5 signalling in cancers, there are several STAT5 inhibitors that can prevent STAT5 phosphorylation, dimerisation, or its transcriptional activity. Tyrosine kinase inhibitors (TKIs) that target molecules upstream of STAT5 could also be utilised. Consequently, since STAT5 contributes to tumour aggressiveness and cancer progression, inhibiting STAT5 constitutive activation in cancers that rely on its signalling makes for a promising targeted treatment option.

## 1. Introduction

The signal transducer and activator of transcription (STAT) family is comprised of seven members, namely STAT1, STAT2, STAT3, STAT4, STAT5a, STAT5b and STAT6 [1,2,3,4]. These proteins are transcription factors that are activated upon phosphorylation by Janus kinases (JAKs) in response to cytokine signalling [1,5]. Upon the binding of a cytokine to its receptor, the activated receptor on the cell surface membrane will induce the phosphorylation of JAKs, which will recruit the corresponding STAT protein to activate it by phosphorylation [6,7,8,9]. Full activation of the phosphorylated STAT proteins only occurs when they either homo- or heterodimerise, upon which they could form stable tetramers that translocate to the nucleus to bind more efficiently to the interferon gamma activated sites (GAS) of the promoters of STATs-regulated genes [5,7,10,11,12,13]. Apart from its activation, the STAT signalling pathway could also be negatively regulated by other proteins, such as phosphatases, the suppressors of cytokine signalling (SOCS) and protein inhibitors of activated STAT (PIAS), which dephosphorylate activated JAKs and STATs, prevent STATs activation by JAKs, and bind activated STATs to GAS sites, respectively [5,14,15,16].

Several STAT proteins have been found to be linked to the cancer pathology, for example, constitutively activated STAT1, STAT3 and STAT5 have been found in breast, lung, prostate and pancreatic cancers, and other haematological malignancies [17,18,19,20,21,22]. The upregulation of these STATs signalling pathways promotes tumour growth and survival due to the inhibition of apoptosis, increased cell proliferation, migration and invasion, and dysregulated immune surveillance [17,23,24,25]. Of these three STAT proteins that are most implicated in cancer, many studies have been focused on the roles of STAT3 in tumour development [17,26,27,28]. However, it was only more recently that STAT5 has been shown to play a major role in the tumour progression of several cancers as well. Therefore, we will be focusing on the implications of constitutive STAT5 signalling in various cancers, as well as the therapies available to target it.

## 2. STAT5 and Its Isoforms

STAT5 was originally discovered as a transcription factor of the β-casein gene in lactating mammary cells and was called the mammary gland factor (MGF) [29]. The cloning of MGF indicated that it belonged to the STAT family of proteins and was then designated as STAT5 [30]. It is a protein of 794 amino acids, and could be activated by prolactin (PRL) signalling through JAK2 phosphorylation on its Tyr694 residue [29,30].

**Figure 1:**
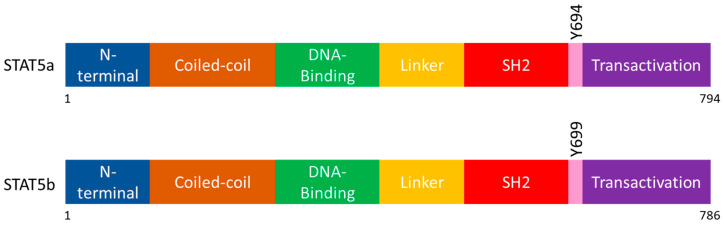
Structure of the STAT5 isoforms, STAT5a and STAT5b.

Further molecular studies have elucidated that two clustered genes on chromosome 17 in humans encode for STAT5. The two genes give rise to two different isoforms, STAT5a and STAT5b, which have more than 90% peptide sequence identity [31]. STAT5a was used to denote the original STAT5 discovered in PRL signalling, while STAT5b is a protein with 786 amino acids, and is phosphorylated on its Tyr699 residue in contrast to Tyr694 in STAT5a [31]. Both isoforms share the same functional domains arrangement, as shown in Figure 1, and have similar structures. Since the two isoforms are highly similar, they have redundant functions in regulating genes responsible for some cellular processes, such as cell proliferation and apoptosis [32]. However, due to structural differences on the C-terminal regions, they also have non-redundant functions. For example, STAT5a and STAT5b have been found to be associated with genes modulating neural and T-cell development, respectively [32].

## 3. Roles of STAT5 in Physiology

**Figure 2:**
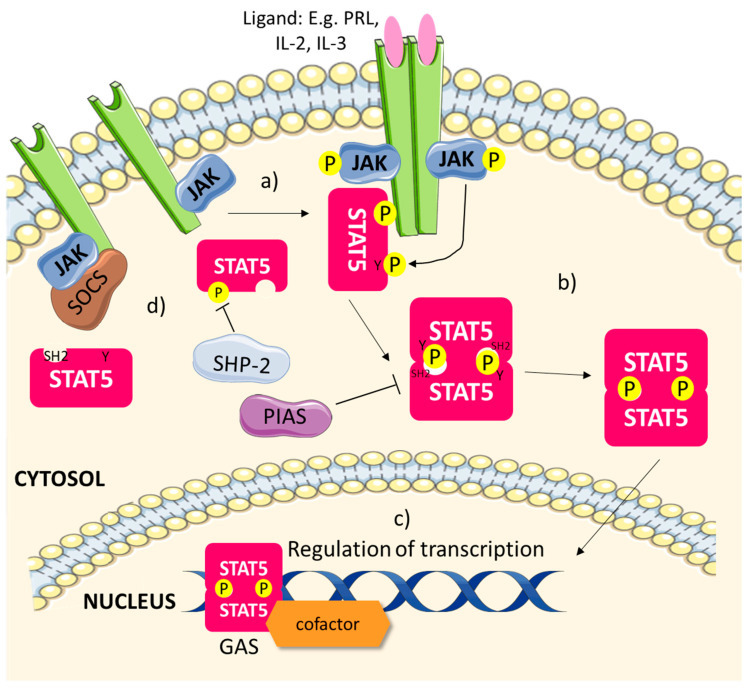
Activation of JAK/STAT5 signalling. (**a**) Upon the binding of ligands (e.g., cytokines) to their respective receptors, the receptors dimerise, allowing JAKs to phosphorylate each other as well as the receptor. Free STAT5 monomers in the cytosol are recruited to the receptor and are phosphorylated on their tyrosine residue by JAKs. (**b**) Phosphorylated STAT5 dimerises; the SH2 domain of one STAT5 interacts with the phospho-tyrosine (pY) region of the reciprocal STAT5. (**c**) The STAT5 dimer translocates into the nucleus and binds to interferon gamma activated sited (GAS) elements, recruiting cofactors, such as bromodomain and extra-terminal domain (BET) family proteins, to regulate the transcription of its target genes, for example cyclin D and the serine/threonine kinase Pim-1. (**d**) Negative regulators of the JAK/STAT5 pathway, tyrosine phosphatase SHP-2, suppressor of cytokine signalling (SOCS) and protein inhibitors of activated STAT (PIAS), could modulate the activation of the pathway.

The activation of STAT5 (Figure 2) occurs when ligands responsible for this signalling pathway, such as the cytokines interleukin-2 (IL-2) and IL-3, bind to their respective receptors, causing them to dimerise or multimerise, bringing JAKs into close proximity to transphosphorylate each other as well as the receptors [14,33,34,35]. This will recruit STAT5 to the receptors, and they will be phosphorylated on their critical tyrosine residue by the activated JAKs [34,35]. Once they are phosphorylated, STAT5 will dimerise with each other, where the SH2 domain of each STAT5 molecule will interact via the phospho-tyrosine (pY) residue of the other STAT5 [14,34,35]. The translocation of the fully activated STAT5 dimer into the nucleus will then occur, whereby it will bind to GAS elements through its DNA-binding domain (DBD) [35,36]. Transcriptional regulation of STAT5 target genes, such as those that promote cell proliferation, cyclin D and serine/threonine kinase Pim-1, as well as those involved in apoptosis like Janus kinase-binding protein (JAB), could then be activated by the DNA-bound STAT5 dimer through its transactivation domain (TD) [36]. The transcriptional activities of STAT5 are also modulated by the presence of other transcription cofactors/activators, e.g., the bromodomains and extra-terminal domain (BET) family of bromodomain-containing proteins and centrosomal P4.1-associated protein (CPAP), as well as cell-specific transcription factors, such as the glucocorticoid receptor (GR) and CCAAT/enhancer binding protein (C/EBP) in adipocytes [34,37,38]. Other factors that could affect the activation of the JAK/STAT5 signalling pathway include its negative regulators, tyrosine phosphatase SHP-2, SOCS and PIAS [14,35]. SHP-2 could dephosphorylate activated STAT5, while SOCS could bind to JAKs and the receptors to inhibit STAT5 phosphorylation [14,35]. PIAS, on the other hand, could prevent the activation of STAT5 via disrupting its dimerisation, thus STAT5 would be unable to translocate into the nucleus to bind to GAS sites [14]. These key players in the JAK/STAT5 pathway play a central role in regulating STAT5 activity physiologically.

STAT5 signalling is important for the development of normal mammary glands and lactation during pregnancy. STAT5a activation, in particular, promotes alveolar cell proliferation and differentiation during lactation [39]. STAT5a deficiency resulted in the incomplete maturation of the mammary gland, which was accompanied by a reduced number of alveoli and no milk production in post-partum mice [39]. The effects of STAT5a deficiency could partially, but not fully, be compensated by the activity of STAT5b [40]. Moreover, STAT5a also functions to promote the survival of mammary epithelial cells [40].

STAT5 also plays a crucial role in the regulation of haematopoiesis. A study done by Nosaka et al. showed that STAT5 is responsible for the proliferation, differentiation and apoptosis of haematopoietic cells in response to IL-3 signalling [41]. STAT5 activation induced the transcription of Pim-1 independently of IL-3 signalling, promoting cell proliferation. However, in the presence of IL-3, p21 and JAB are transcribed in response to STAT5 activation to cause the differentiation and apoptosis of the cells, respectively [41]. IL-2-induced STAT5 signalling limits the T helper 17 (Th17) cell population by inhibiting the production of IL-17 [42]. In addition, STAT5 functions to modulate Forkhead box protein P3 (FoxP3) for the generation of regulatory T cells [43]. STAT5b is also involved in the antigen re-stimulation T cell death (RICD) of effector memory T cells, which helps to maintain T cell homeostasis in the body [44]. Typically, STAT5 and STAT3 regulate different genes in cells where they both might be activated, but in some cases, they could also regulate the expression of the same gene [34]. In both Th1 and natural killer (NK) cells, STAT3 activates the transcription of B-cell lymphoma protein 6 (Bcl6), which in turn is suppressed by STAT5 in response to IL-2 [45]. In this instance, STAT5 binding to the promoter of the *Bcl6* gene is dominant over STAT3 as it could displace the DNA-bound STAT3 to repress the transcription of Bcl6 [46]. Hence, STAT5 signalling is necessary to determine the lineages of T cells.

Moreover, several studies using knockout (KO) mice models have elucidated both the redundant and non-redundant functions of the STAT5 isoforms physiologically. These KO mice models were generated by injecting mouse embryonic stem (ES) cells that had individual or both isoforms of STAT5 successfully targeted into the blastocysts of 129 × BALB/c and C57BL/6 mice strains [47,48,49]. STAT5a KO mice did display a defect in the development of the mammary gland, which was not seen in STAT5b KO mice [47,49]. On the other hand, STAT5b KO mice lost sexual dimorphism in response to growth hormone signalling, which affects both their growth rates as well as the expression of liver genes, such as the major urinary proteins (MUP) [47,48,49]. Early post-natal lethality was observed in a third of STAT5a/b double KO mice [49]. Both STAT5a/b isoforms also contribute to the fertility of female mice, whereby STAT5a/b KO, but not individual KOs, mice were infertile due to a loss of the expression of cytokine-inducible SH2-containing protein (CIS), a STAT5 regulated gene, in the ovaries [49]. Both STAT5 isoforms are also important in regulating body growth rates and haematopoiesis, which is discussed above, as double KO mice were smaller in size and had a reduced lymphocyte population, compared to the wild type or individual KOs mice [49].

## 4. Roles of STAT5 in Various Cancers

**Figure 3:**
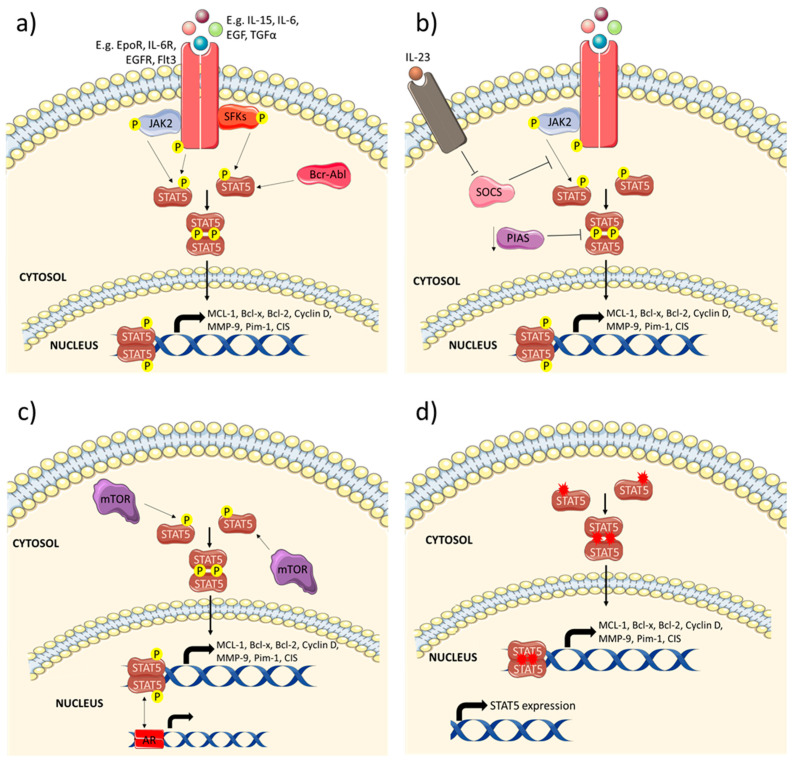
Mechanism of constitutive activation of STAT5. STAT5 is activated by: (**a**) increased signalling by cytokines and their receptors (interleukin-15 (IL-15), IL-6, erythropoietin receptor (EpoR), and IL-6 receptor (IL-6R)), receptor tyrosine kinases (RTKs), e.g., epidermal growth factor receptor (EGFR), and Flt3, as well as non-receptor tyrosine kinases (nRTKs), such as Src family kinases (SFKs), JAK2 and Bcr-Abl; (**b**) downregulation of negative regulators of STAT5 pathway, e.g., SOCS inhibited through IL-23 signalling and PIAS; (**c**) cross-talk with other signalling pathways, e.g., mammalian target of rapamycin (mTOR) and androgen receptor (AR) pathways; and (**d**) activating STAT5 mutations and increased STAT5 expression.

In recent years, there has emerged increasing evidence of the importance of STAT5 in cancer development. Aberrant STAT5 signalling, mostly due to its constitutive activation (Figure 3) or due to the loss of STAT5, has been found to drive tumour survival, growth, metastasis and resistance to anti-cancer therapies. It contributes to the pathology of various cancers (summarised in Table 1), such as breast, colorectal, lung, prostate and liver cancer, and haematological malignancies, which are amongst the top 10 cancers with the highest mortality rates [50].

### 4.1. Breast Cancer

Active STAT5 has been detected across all types of breast cancers, in oestrogen receptor (ER)-positive, HER2-positive, and triple-negative breast cancer (TNBC) [51]. Various studies have shown that STAT5 could act as both a tumour suppressor and an oncogene in breast cancer under different circumstances. As discussed previously, STAT5 activity is likely to be dominant over that of STAT3. Hence, as a tumour suppressor, STAT5 activation can counteract the oncologic effects of STAT3, which is more commonly constitutively activated in breast cancer compared to STAT5 [51]. STAT5 signalling in tumour cells with active STAT3 reduced their proliferation and also sensitised the cells to treatment with paclitaxel and vinorelbine [51]. In ER-positive breast cancer, STAT5 expression enhanced the response to hormone therapy and increased the overall survival of patients [84]. Statistical analysis of patients’ data also elucidated the association of STAT5 with increased breast cancer-specific and disease-free survival, especially in lymph node-negative breast cancer [85]. Furthermore, STAT5 is also progressively inactivated with the progression to metastatic breast cancer due to enhanced regulation by tyrosine phosphatases, such as protein tyrosine phosphatase 1B (PTP1B) [52,85]. The activation of STAT5 in breast cancer cells could also inhibit their migratory and invasive potential through the downregulation of matrix metalloproteases (MMP) 2 and 9, while upregulating E-cadherin expression on the cell surface [53]. STAT5 could also reduce the expression of activator protein 1 (AP-1), a protein that promotes cell survival, proliferation, angiogenesis and invasion [54].

However, in mice models, increased STAT5 signalling induces the formation of tumours in the breast more rapidly. Overexpression of transforming growth factor-α (TGFα), which enhances epidermal growth factor receptor (EGFR) activation, promoted the development of hyperplasia and tumours about one and a half months earlier in STAT5-expressing mice compared to STAT5a KO mice [40]. Hence, the activation of STAT5a also contributes to the initiation of breast cancer, which is an opposing trend from the tumour-suppressive effects of STAT5. The parity-dependent effects of dysregulated STAT5 signalling in breast cancer are likely to be the reason for these trends. STAT5 is increasingly activated during pregnancy and early lactation stages, while being progressively deactivated in the late lactation and involution stages [86]. Therefore, the constitutive activation of STAT5 could initiate tumorigenesis latently in parity cycles by overcoming the tumour-suppressive effects of pregnancy [55]. Pregnancy causes the differentiation of luminal progenitor cells, resulting in a more differentiated and less proliferative population of cells in the mammary glands [55,87]. However, since STAT5 signalling could increase cell proliferation, its constitutive signalling then prevents the decrease of the luminal progenitor cell population, allowing the mammary glands to be more susceptible to tumorigenesis [55,88]. STAT5 could also induce chromatin structure remodelling and the overexpression of cyclin D1 to enhance tumour formation [55]. The tumours that arise from these instances are usually luminal type-like and are well-differentiated [56]. This suggests that STAT5 may act as an oncogene during the tumorigenesis process, but acts as a tumour suppressor in the early, node-negative stage of the disease, since it is gradually inactivated during cancer progression [51,56].

Moreover, induced Jak2/STAT5 signalling by phosphoinositide 3-kinase/protein kinase B (PI3K/Akt) targeted therapy in TNBC facilitates resistance to the treatment. A combined inhibition of both PI3K/Akt and STAT5 pathways significantly decreased tumour growth and metastasis [57]. Bone metastasis from primary TNBC tumours could also be enhanced by STAT5 signalling, in tandem with Yes-associated protein/transcriptional coactivator with PDZ-binding motif (YAP/TAZ) activation, in response to ABL kinases activity through the upregulation of MMP-1 and IL-6 [58]. STAT5 activation by FYN, a Src family kinase (SFK), also promoted the metastatic abilities of TNBC cells [59]. Additionally, the enhanced transcriptional activity of STAT5b by breast tumour kinase (Brk) through signal-transducing adapter protein 2 (STAP-2) mediation elevated tumour cell proliferation [60]. JAK2/STAT5 signalling has also been shown to increase the expression of heat shock protein 90 alpha (HSP90α) in breast cancer cells, inhibiting apoptosis and enhancing cell survival [61]. Thus, it is likely that in subsets of breast cancer, such as in TNBCs and cancers with TGFα/EGFR activation, STAT5 could act as an oncogene and promote the progression of the disease. In addition, when STAT3 is not concurrently activated with STAT5 (which make up 7% of breast cancer tissues), STAT5 may also act as an oncogene [51]. However, since the activation status of STAT3 was not explored in the above-mentioned studies, a deeper understanding of the role of STAT5 as an oncogene in breast cancer is needed for a more specific targeting of STAT5. For example, the inhibition of overactive STAT5 may prevent the initiation of tumorigenesis, as well as breast cancer progression of certain TNBCs.

### 4.2. Colorectal Cancer

The overexpression and constitutive activation of STAT5 leads to a poorer prognosis of colorectal cancer (CRC). Compared to the normal colon epithelium, CRC tissue has a much higher expression of STAT5 [62,63]. The presence of phosphorylated STAT5 in the cytoplasm of tumour cells also correlates with shorter overall survival [64]. The constitutive activation of STAT5 in CRC could be initiated by IL-23 signalling that inhibits the expression of SOCS3, which functions to inhibit STATs activation [65]. STAT5 activity suppresses p16, p21 and p27, while promoting cyclin D1, Bcl-2 and survivin expression, which enhances CRC cell proliferation and survival and prevents apoptosis [62,63,66]. It also upregulates C9orf140, an Axin1-interacting protein, phospho-focal adhesion kinase (p-FAK), vascular endothelial growth factor (VEGF) and MMP-2 levels, whilst downregulating E-cadherin expression, raising the invasive potential and metastasis of CRC cells [62,67]. The knockdown of STAT5 in CRC cells inhibits cell growth by inducing G1 phase cell cycle arrest and apoptosis [62,63]. A limitation to the current treatment against CRC is the development of resistance to the widely-used chemotherapeutics cisplastin and 5-fluorouracil. STAT5 inhibition could restore the sensitivity of CRC cells to these treatments [68]. Therefore, STAT5 functions to promote tumorigenesis and tumour aggressiveness in CRC, and its inhibition might be important in treating advanced cancer.

### 4.3. Lung Cancer

STAT5 is found to be overexpressed in the cytoplasm and nucleus of tumour cells of various non-small cell lung cancer (NSCLC) subtypes, such as squamous cell carcinoma, adenocarcinoma and large cell carcinoma [70]. STAT5 overexpression is most prominent in pT2 size NSCLC tumours [89]. Furthermore, nuclear STAT5 is associated with the nuclear Bcl-xL expression of large cell carcinoma, suggesting that STAT5 could regulate the pathogenesis of lung cancer through Bcl-xL, which is correlated with distant metastasis of the cancer [70]. There is also a correlation between STAT5 and cyclooxygenase-2 (COX-2) expression in NSCLC tissues [89]. Epidermal growth factor (EGF) signalling could activate STAT5 to promote COX-2 expression, which could enhance disease progression through inflammation [90]. STAT5 activation in NSCLC is mediated by IL-6 signalling, JAK1, JAK2 and c-Src, as well as a downregulation of PIAS3 [71,89]. However, the effects of inhibiting STAT5 activation in NSCLC cells decreases cell proliferation, and increases G1 phase cell cycle arrest and apoptosis [71], suggesting a potential with regards to targeting STAT5 for therapeutics in NSCLC.

### 4.4. Prostate Cancer

In prostate cancer, the activation of STAT5 is associated with high histological grades, early disease recurrence and shorter progression-free survival [91]. STAT5 constitutive activation is found in a majority of prostate cancers, but not in normal epithelium, and is most significant in recurrent prostate cancer [72,92]. STAT5 phosphorylation in prostate cancers can be mediated by JAK2 as well as the Erythropoietin receptor (EpoR) for STAT5b [73,93]. The androgen receptor (AR) could also work synergistically with STAT5 for them to enhance each other’s transcriptional activity, which increases the expression of both their target genes [92]. Additionally, STAT5 activity stabilises AR from proteasomal degradation, inducing the progression of prostate cancer to castration-resistance [94]. A study has also found that the STAT5 transcriptional targets in prostate cancer consist of 21%, 8% and 4% of metastatic-, proliferation- and apoptosis-related genes, respectively [74]. Moreover, STAT5 knockdown in prostate cancer has also been shown to inhibit tumour growth and induce apoptosis [72,73]. Hence, STAT5 promotes tumour progression in prostate cancer by increasing cell proliferation and metastasis, and inhibiting apoptosis.

### 4.5. Hepatocellular Carcinoma (HCC)

Similar to breast cancer, STAT5 also has a dual role in HCC, where it can act as a tumour suppressor to counteract the tumorigenic effects of STAT3 signalling or as an oncogene in other circumstances. According to a study by Hosui et al., STAT5 inhibits tumour formation by balancing the levels of STAT5 and STAT3 dependent-signalling pathways [75]. With the loss of STAT5, mature TGFβ is stabilised, resulting in an increased activation of STAT3, enhancing liver fibrosis and HCC development [75]. Additionally, another study has elucidated that STAT5 could also act as a tumour suppressor by upregulating the expression of NADPH oxidase 4 (NOX4), an enzyme involved in the generation of reactive oxygen species (ROS), as well as that of the p53 upregulated modulator of apoptosis (PUMA) and the Bcl-2-interacting mediator of cell death (BIM), which are pro-apoptotic proteins [76].

However, STAT5 could also promote cancer development and progression by enhancing cell proliferation, cancer stem cells (CSCs) population, chemoresistance and epithelial–mesenchymal transition (EMT), a key precursor phenotype of tumour cells that could lead to increased invasion and metastasis [77,78,79]. Direct STAT5 phosphorylation by mammalian target of rapamycin (mTOR) could modulate the expression and nuclear localisation of sterol regulatory element binding protein-1 (SREBP1) to promote lipid synthesis in the liver, which could result in the onset of HCC [95]. Moreover, increased STAT5 expression in HCC regulated by GRAM domain-containing 1A (GRAMD1A), a cholesterol transporter, could induce tumour growth, increased CSCs side population, chemoresistance and tumour cell survival by upregulating the expression of cyclin D1, Bcl-2, c-Myc and c-Jun, as well as downregulating caspase 3 and poly (ADP-ribose) polymerase (PARP) [77]. Consistent with the roles of STAT5 in tumour progression, STAT5b expression has also been associated with advanced tumour stages and poor survival in HCC patients [79]. Insulin-like growth factor 1 (IGF-1) and Hepatitis B protein, HBx, could enhance STAT5b signalling, leading to the increased migratory and invasive capabilities of HCC cells by promoting EMT [78,79]. This activation of STAT5 stimulates the EMT of HCC cells through the downregulation of E-cadherin, while upregulating the expression of N-cadherin and Vimentin [78,79]. Hence, the contributions of STAT5 activation in HCC share certain similarities with breast cancer—STAT5 can act both as a tumour suppressor, in conditions where STAT3 is active in tumorigenesis, or as an oncogene to drive tumour aggressiveness in other instances.

### 4.6. Haematological Malignancies

STAT5 is especially important in the pathology of many haematological cancers, since it is a key molecule in the regulation of haematopoiesis. It is often constitutively activated in various leukaemic types, such acute/chronic myeloid leukaemia (AML/CML), acute lymphoblastic leukaemia (ALL) and peripheral T-cell lymphoma (PTCL). For example, STAT5 is constitutively activated in 70% of AML patients, and is required to maintain the disease state in Bcr-Abl-initiated ALL and CML [80,96]. Unlike in solid tumours, mutations in STAT5 are more common in driving its constitutive activation in the various haematological cancers [97]. Kollman et al. discovered that somatic mutations in STAT5b, such as the SH2 domain mutation of STAT5b N642H, inhibited interferon-α/γ (IFNα/γ) signalling in Bcr-Abl-driven leukaemia, thus promoting tumour formation and growth [81,98]. These mutations occur more frequently in STAT5b than in STAT5a, and STAT5b was also elucidated to play a more prominent role in the initiation and progression of these malignancies than STAT5a [81].

In addition, STAT5 can be activated through phosphorylation by type 3 receptor tyrosine kinase Flt3, JAK2 and Bcr-Abl, as well as in response to IL-15 signalling [80,82,99,100,101]. In myeloid leukaemia, the Bcr-Abl oncogene could induce the phosphorylation of STAT5 through the mediation of Hck, a member of the SFK [102]. STAT5 signalling upregulates the expression of its target genes, A1, Pim-1 and cyclin D2, promoting cell proliferation and leukemogenesis [101,102]. DNA repair proteins, Ataxia telangiecstasia mutated (ATM) and tumour protein p53-binding protein 1 (TP53BP1), are also downregulated in response to STAT5 signalling [101]. Moreover, phosphorylated STAT5 could interact with PI3K to activate the PI3K/Akt pathway, enhancing cell growth [103]. On the other hand, the inhibition of STAT5 reduces anti-apoptotic proteins, Bcl-2 and MCL-1, as well as increasing pro-apoptotic protein, Bim, expression [104]. Therefore, constitutive STAT5 activation in leukaemia can result in increased cell transformation, proliferation, survival, drug resistance to imatinib and the inhibition of apoptosis [80,82,83].

## 5. Targeting STAT5 in Cancer

As STAT5 has been shown to be involved in the initiation and progression of several cancers, the inhibition of STAT5 activity in the types of cancers discussed above, as well as in STAT3-inactive breast and liver cancers, could prove to have therapeutic benefits for the patients. The constitutive activation of STAT5 signalling in cancer can be abrogated by STAT5 inhibitors (Table 2) or tyrosine kinase inhibitors (TKIs) (Table 3) that target the upstream signalling molecules, such as JAK, Flt3 and Bcr-Abl. The administration of STAT5 inhibitors in combination with the TKIs could promote synergistic therapeutic effects.

### 5.1. STAT5 Inhibitors

The activation of STAT5 first occurs with it being phosphorylated on its critical tyrosine residue, before it dimerises with the help of its SH2 domain. Following that, STAT5 could translocate to the nucleus and act as a transcription factor to promote the transcription of its target genes. STAT5 inhibitors that could inhibit any of the key stages in its activation could prove to be a novel treatment against cancers that are dependent on STAT5 signalling in their pathogenesis. There are currently several classes of inhibitors that can interact with STAT5 to inhibit its activity. One class of inhibitors prevents the tyrosine phosphorylation of STAT5, which is required for its activation. Another class targets the SH2 domain of STAT5, thus blocking it from being able to dimerise and achieve full activation. The third class of STAT5 inhibitors can bind to transcriptional regulator Bromodomain Containing 2 (BRD2), inhibiting STAT5-BRD2 interaction, and thus attenuating the transcriptional activity of STAT5.

#### 5.1.1. STAT5 pY Inhibitor

A study by Nelson et al. has identified pimozide as a potential STAT5 inhibitor to treat CML. Pimozide is an antipsychotic drug that is currently in use for the treatment of Tourette syndrome. However, a high throughput screen elucidated that pimozide is a potent inhibitor of STAT5 that can halt its transcriptional activity, but not that of NF-κB and STAT1 [105]. It was found that pimozide can inhibit the critical tyrosine residue phosphorylation, which is needed to activate STAT5, when administered to established CML cell lines, KU812 and KU562, but has non-significant effects in the inhibition of Bcr-Abl or other SFKs [105]. Hence, it was postulated that pimozide can directly act on STAT5 without it being a tyrosine kinase inhibitor. Pimozide also has negligible effects on the induced phosphorylation of STAT1 and STAT3 [105]. By preventing the tyrosine phosphorylation of STAT5, pimozide can decrease the expression of STAT5 target genes in KU812 and KU562 cells, such as Bcl-x, Pim-1, CIS, MCL-1 and cyclin D1, and reduce cell viability as well as induce apoptosis of the tumour cells [105]. Furthermore, pimozide was also discovered to be effective in the treatment of CML cells that are resistant to imatinib, the first-in-line treatment for CML that is driven by Bcr-Abl [105]. The combination treatment of both pimozide and a TKI also results in a synergistic effect, which increases the efficacy of the treatment against CML [105]. Similar results were also shown in AML cells driven by Flt3 mutations, where a combination of pimozide and a TKI, PKC412 or sunitinib exhibited strong synergistic effects [106]. Pimozide can also overcome the resistance that Flt3 confers to AML cells to treatment with PI3K/Akt inhibitors, by attenuating STAT5 activation, which in turn prevents the activation of mTOR and decreases the expression of MCL-1 [107]. Pimozide treatment alone in an AML mouse model resulted in reduced tumour burden and increased the survival of the mice in comparison to the control group, with no significant changes to the body weight, which showed that pimozide’s efficacy and safety in vivo [106]. Another study also elucidated that pimozide is effective in decreasing STAT5 PTCL, a type of aggressive heterogenous non-Hodgkin’s lymphoma [108]. Pimozide treatment induced the apoptosis of PTCL cells by activating the extrinsic apoptotic pathway through TNF-related apoptosis-inducing ligand/death receptor 4 (TRAIL/DR4) [108]. Hence, pimozide could prove to be an efficient way to target haematological malignancies dependent on STAT5 signalling by inhibiting its tyrosine phosphorylation.

Despite the promising data on haematological malignancies, the mechanism of action of pimozide is still not clearly understood. Other studies have shown that pimozide treatment could also reduce Wnt/β-catenin signalling and STAT3 phosphorylation in other types of cancer, such as HCC and prostate cancer [109,110,111]. It is thus possible that STAT5 pY inhibition by pimozide could be contributed through its effects on other signalling pathways, and it does not target STAT5 specifically. Therefore, further investigations into pimozide’s mechanism of action to determine the predominant pathway that it inhibits, as well as its target specificity in various types of cancers, are needed.

#### 5.1.2. SH2 Domain Inhibitors

Over the past few years, several novel compounds have been discovered, and these can bind to the STAT5 SH2 domain to block the full activation of STAT5 molecules (Table 2). Since the homology of the SH2 domain of STAT5 is significantly different from that of STAT1 and STAT3, the novel compounds are specific in targeting STAT5 activation [112,113]. The first non-peptide small compound that was found to block the SH2 domain of STAT5 is a chromone-based acyl hydrazine, nicotinoyl hydrazine [114]. It could attenuate the phosphorylation of STAT5, thus preventing it from binding to DNA, but has negligible effects on STAT1 and STAT3 phosphorylation [114]. In breast cancer, nicotinoyl hydrazine significantly reduced the proliferation of T47D cells, albeit at high concentrations of 200 and 400 μM [115].

Other compounds subsequently found to have SH2 domain inhibitory activity are salicylic acid-derived compounds, such as BP-1-108, 13a and AC-4-130 [112,113,116]. The administration of BP-1-108 to MV-4-11 AML and K562 CML cell lines reduced STAT5 phosphorylation and the expression of target genes, such as cyclin D, MCL-1 and Myc [113]. This promoted the increased apoptosis of these cancer cells. In addition, higher concentrations of BP-1-108, reaching up to 160 μM, did not induce significant adverse effects on healthy bone marrow cells, which is about four to eight times more concentrated than its IC_50_ in K562 and MV-4-11 cells, respectively [113]. The compound 13a was shown to be highly specific towards STAT5, being seven times more selective for STAT5 than STAT3 [116]. It also had negligible effects on other kinases, such as JAK1/2, Abl and Flt3 [116]. Similar to BP-1-108, 13a could reduce the expression of phosphorylated STAT5 and its target genes in MV-4-11 AML cells, thus inducing their apoptosis [116]. The most recently identified salicylic acid-based compound, AC-4-130, could inhibit STAT5 dimerisation in AML cells, resulting in decreased STAT5 phosphorylation as well as nuclear translocation [112]. This compound, resembling the others above, also reduced the expression of STAT5 target genes, which led to decreased cell viability, cell cycle arrest in the G0/G1 phase and apoptosis of AML cells. Moreover, AC-4-130 also attenuated the proliferation of AML CSCs [112]. In the in vivo AML xenograft mice model, 25 mg/kg of AC-4-130 could inhibit AML development and tumour growth, while not having any significant adverse effects on healthy blood cells [112]. This SH2 domain inhibitor could also sensitise AML to other TKI inhibitors, so it has the potential to be developed as a combination therapy with TKIs in the future [112]. However, since the research on various SH2 inhibitors is still in early stages and is mostly focussed on haematological malignancies, more investigations are required to validate their efficacy and safety for the treatment of other cancers dependent on STAT5 signalling both in vitro and in vivo.

#### 5.1.3. STAT5 Transcriptional Activity Inhibitors

Other transcriptional cofactors are required to interact with STAT5 to promote the transcription of its target genes optimally. Thus, inhibiting the interaction between STAT5 and its transcriptional cofactors could prevent the transcriptional activation of target genes mediated by STAT5. One example of a cofactor assisting STAT5 transcriptional activity is BRD2, a transcriptional regulator of the BET family of proteins [38]. An inhibitor that could inhibit the interaction between STAT5 and BRD2 could also reduce STAT5 transcriptional activity. One such inhibitor is JQ1, which could decrease the expression of STAT5 target genes, such as Bcl-x, PIM and CIS, in ALL cells, but not those of STAT3 [38,94]. The use of JQ1 also reduced the cell viability of both established ALL cell lines as well as primary ALL cells, especially when it was administered in combination with other TKIs [38,111].

Another type of inhibitor that can reduce STAT5 transcriptional activity is a compound that can block the DBD of the STAT5 molecule to prevent it from binding to GAS elements of the promoter of its target genes. A study by Wang et al. showed that a 21-mer decoy oligodeoxynucleotides (dODN) could attenuate STAT5 activity in CML, reducing the expression of STAT5 target genes and thus decreasing cell viability and inducing G0/G1 phase cell cycle arrest and apoptosis. The dODN is specific in targeting the STAT5 signalling pathway through its specificity towards STAT5a and STAT5b, but not STAT3 [117]. As with the SH2 inhibitors, these compounds that target STAT5 transcriptional activity are in their early stages of validation, and additional in vivo testing is required to ascertain their efficacy and safety for haematological malignancies. The effects of these STAT5 transcriptional activity inhibitors should also be screened in solid cancers driven by STAT5 to determine if they could be viable treatment options for these cancers in the future.

### 5.2. Tyrosine Kinase Inhibitors

Since STAT5 activation in the various cancers is dependent on the phosphorylation by various tyrosine kinases, for example JAK2, Bcr-Abl, Flt3 and Src, targeting the tyrosine kinases to inhibit the activation of STAT5 is a feasible way of treating cancers dependent on STAT5 signalling. There are several different TKIs that are Food and Drug Administration (FDA) approved for the treatment of CML, which is often dependent on STAT5 signalling, such as imatinib that inhibits Bcr-Abl, dasatinib and nilotinib [118,119]. However, over time a significant number of patients treated with TKIs become resistant to the treatment [118]. As such, other TKIs have to be developed as second-in-line or third-in-line therapies to overcome the resistance, such as some of those listed in Table 3. Another option is to administer a combination treatment of several TKIs or a TKI with a STAT5 inhibitor, which could display a synergistic effect and combat the resistance problem. For example, AC-4-130, a STAT5 SH2 domain inhibitor, showed synergistic effects with Ruxolitinib, whereby the low concentration of 1µM for both compounds could reduce the cell viability of MV4-11 and MOLM-13 AML cell lines to below 50% in 24h, which was not observed when the compounds were administered separately [112]. Further screening showed that AC-4-130 could have possible synergistic effects with dasatinib as well [112,115].

## 6. Conclusions

Under physiological conditions, STAT5 is required for the proper development of the mammary gland and the immune system. However, its activation in various cancers is mostly associated with disease progression and poor prognosis. Inhibiting the STAT5 signalling pathway with STAT5 inhibitors in combination with various TKIs could be a promising targeted treatment option for cancers driven by constitutively activated STAT5. Since most STAT5 inhibitors are not yet extensively validated for their safety and efficacy in the treatment of cancer patients, and their studies have mostly been focused on targeting haematological malignancies, more research is required to develop novel therapies for STAT5-dependent cancers. Additionally, since STAT5 could also act as a tumour suppressor in certain subtypes of breast cancer and HCC, and as an oncogene in others, a clearer distinction between the different circumstances under which either function of STAT5 is a resulting effect of its signalling is necessary. This is so as to be able to target the oncogenic constitutive activation of STAT5 more accurately and effectively in these cancers, whilst not inhibiting its activation when STAT5 is exerting its tumour-suppressive effects. Ergo, although studies have shown that STAT5 is highly involved in oncogenic processes, there is still a need for further investigations into its roles for the development of effective therapies against STAT5.

## Figures and Tables

**Table 1:** The role of STAT5 as either a tumour suppressor or an oncogene in various cancers, and its effects in promoting cancer progression.

Types of Cancer	Role of STAT5	STAT5 Status	Effects of the Status STAT5 in the Various Cancers	Ref.
**Breast**	Tumour suppressor	Inactivated and loss of expression	↑ STAT3 signalling ↑ Metastasis More aggressive cancer	[51,52,53,54]
	Oncogene	Constitutively activated	↑ Tumour initiation ↑ Drug resistance, metastatic capabilities of TNBC	[40,55,56,57,58,59,60,61]
**Colorectal**	Oncogene	Overexpressed and constitutively activated	↑ Cell proliferation, survival, metastasis, and drug resistance ↓ Apoptosis	[62,63,64,65,66,67,68,69]
**Lung**	Oncogene	Overexpressed	↑ Cell proliferation and metastasis	[70,71]
**Prostate**	Oncogene	Constitutively activated	↑ Cell proliferation and metastasis ↓ Apoptosis	[72,73,74]
**Liver**	Tumour suppressor	Loss of expression	↑ STAT3 signalling leading to development of HCC. ↓ Apoptosis	[75,76]
	Oncogene	Increased activation	↑ Cell proliferation, metastasis, drug resistance, and CSC population	[77,78,79]
**Haematological malignancies**	Oncogene	Mutated and constitutively activated	↑ Initiation of cancer ↑ Cell proliferation, survival, and drug resistance ↓ Apoptosis	[80,81,82,83]

Abbreviations: ↑ increase; ↓ decrease; CSC cancer stem cell; HCC hepatocellular carcinoma; TNBC triple-negative breast cancer.

**Table 2:** A list of selected STAT5 inhibitors.

Target	Drug	Cancer	Effects	Molecular Effects	Ref.
pY phosphorylation	Pimozide	AML	↓ Cell viability ↑ Apoptosis in combination with a TKI		[106]
		CML	↑ Apoptosis ↓ Colony formation	↓ Bcl-x, Pim-1, CIS, cyclin D1, MCL-1	[105]
		PTCL	↓ Cell viability ↑ Apoptosis	↑ Cleaved caspase 8, cleaved caspase 3	[108]
STAT5 SH2 domain	Nicotinoyl hydrazine (CAS 285986-31-4)	Breast	↓ Cell proliferation		[114,115]
	BP-1-108	AML CML	↓ Cell viability ↑ Apoptosis	↓ Cyclin D1, cyclin D2, MCL-1, Myc	[113]
	13a	AML CML	↓ Cell viability ↑ Apoptosis	↓ Cyclin D1, cyclin D2, MCL-1, Myc ↑ Cleaved caspase 3, cleaved PARP	[116]
	AC-4-130	AML	↓ Cell viability ↑ Apoptosis ↓ Growth of CSCs	↓ Cyclin D2, Bcl-2, Myc	[112]
BRD2 / STAT5 transcriptional activity	JQ1	ALL	↓ Cell viability especially in combination with imatinib		[38]
STAT5 DBD / transcriptional activity	21-mer dODN	CML	↓ Cell viability ↑ G0/G1 cell cycle arrest ↑ Apoptosis	↓ Bcl-xL, cyclin D1, c-Myc	[117]

Abbreviations: ↑ increase; ↓ decrease; ALL acute lymphoblastic leukaemia; AML acute myeloid leukaemia; BRD2 Bromodomain Containing 2; CML chronic myeloid leukaemia; CSCs cancer stem cells; DBD DNA-binding domain; dODN decoy oligodeoxynucleotides; PTCL peripheral T-cell lymphoma; TKI tyrosine kinase inhibitor.

**Table 3:** Tyrosine kinase inhibitors (TKIs) that can indirectly target STAT5.

Drug	Target	Cancer	Ref.
Ruxolitinib in combination with nilotinib	JAK2	CML	[120]
Dasatinib (BMS-354825)	Bcr-Abl SFKs	CML ALL	[121]
Lestaurtinib (CEP-701)	JAK2 Flt3	AML	[69]
PD180970	Bcr-Abl	CML	[122]
AZD1480	JAK2	Prostate	[73]
Sunitinib (SU11248)	Flt3	AML	[123]

Abbreviations: ALL acute lymphoblastic leukaemia; AML acute myeloid leukaemia; CML chronic myeloid leukaemia; SFKs Src family kinases.
